# Comparative genomics reveals the molecular basis for divergent algicidal strategies in two *Alteromonas macleodii* strains

**DOI:** 10.1128/aem.01965-25

**Published:** 2025-12-29

**Authors:** Yuxin Lai, Xinyu Liu, Zhiyuan Chen, Yue Li, Xinguo Shi

**Affiliations:** 1 Technical Innovation Service Platform for High Value and High Quality Utilization of Marine Organism, Fuzhou University 12423, Fuzhou, China; 2 College of Advanced Manufacturing, Fuzhou University 12423, Jinjiang, China; 3 College of Biological Science and Engineering, Fuzhou University 12423, Fuzhou, China; 4 Maynooth International Engineering College, Fuzhou University 12423, Fuzhou, China; The University of Arizona, Tucson, Arizona, USA

**Keywords:** algicidal bacteria, harmful algal blooms, genome, quorum sensing

## Abstract

Marine bacteria such as *Alteromonas* are key players in regulating algal blooms, yet the genomic basis for their strain-specific algicidal activities remains poorly understood. Here, we use comparative genomics to dissect the mechanisms of functional divergence between two closely related *Alteromonas macleodii* strains: strain FDHY-03, which employs a broad-spectrum strategy, and FDHY-CJ, which has adapted a narrow-spectrum strategy specifically targeting diatoms. We reveal that these distinct predatory strategies are underpinned by divergent genomic architectures. The broad-spectrum strain FDHY-03 leverages a versatile, synergistic enzymatic arsenal rich in polysaccharide lyases to enable its broad-spectrum attack. In contrast, the specialist FDHY-CJ has evolved an integrated, high-precision system comprising: (i) a specialized CAZyme toolkit, uniquely enriched with GH16 isoforms, tailored to breach diatom-specific defenses; (ii) an enhanced chemotaxis system (Tsr) to home in on its algal targets; and (iii) a complex quorum sensing network (AHL/solo-LuxR) to coordinate its behavior in diatom-rich niches. Our findings provide a high-resolution model for microbial microevolution, demonstrating how genomic plasticity enables rapid niche partitioning within a single species. This work illuminates the molecular details of marine microbial warfare and provides a blueprint for the genome-informed selection of targeted biocontrol agents for harmful algal blooms.

**IMPORTANCE:**

Frequent harmful algal blooms pose a severe threat to global biogeochemical cycles. Algicidal bacteria, acting as natural antagonists, serve as effective biological tools for controlling harmful algal blooms. While extensive research has been conducted on the isolation and identification of algicidal bacteria, the genomic basis for their strain-specific algicidal activity remains unclear. This study employs comparative genomics to analyze the genomic architecture of two closely related *Alteromonas macleodii* strains, revealing distinctly different algicidal strategies. Our findings offer valuable insights into the molecular basis of microbial warfare in marine environments, ultimately contributing to the advancement of microbial-based approaches for mitigating harmful algal blooms.

## INTRODUCTION

The intricate relationship between marine bacteria and phytoplankton governs global biogeochemical cycles and structures marine food webs. Within these dynamic and antagonistic interactions, bacteria lyse bloom-forming algae represent one of nature’s most powerful mechanisms for controlling harmful algal blooms (HABs) (1). As climate change exacerbates the frequency and intensity of HABs, understanding these natural control systems has become a global priority (2). Algicidal bacteria offer a potent, eco-friendly alternative to broad-spectrum chemical treatments, but their effective application hinges on a deep, mechanistic understanding of how they function (3).

The genus *Alteromonas*, a cosmopolitan and metabolically versatile group of Gammaproteobacteria, is a known powerhouse in marine ecosystems, with many strains exhibiting potent algicidal activity (4, 5). However, a critical knowledge gap persists: intraspecific functional diversity. This intraspecific divergence is driven by a highly plastic accessory genome, where mobile genetic elements like plasmids and Flexible Genomic Islands (fGIs) are continuously acquired, exchanged, or lost (6, 7). These elements act as modular toolkits, bestowing strains with unique capabilities for resource utilization (e.g., polysaccharide degradation) (8), environmental resilience (e.g., heavy metal resistance) (9), and ecological interaction through variable cell-surface “glycotypes” that mediate host-phage dynamics (4, 10). While it is clear that different species have different capabilities, the genomic basis for how two closely related strains of the same species can exhibit profoundly different ecological behaviors—one killing a broad range of algae, while another targets only a specific type—remains largely unexplored. This “ecological microdiversity” is the frontier for understanding how bacterial populations adapt and specialize within complex marine environments.

Here, we leverage a natural experiment presented by two *Alteromonas macleodii* strains isolated from HAB events: FDHY-03 functions as a broad-spectrum algicidal predator, whereas FDHY-CJ operates as an integrated, high-precision system specifically adapted to target diatoms (11, 12). Despite their near-identical core genomes, their algicidal phenotypes are divergent. We hypothesized that these distinct lifestyles are driven by strategic differences in their accessory genomes. Using a comparative genomics approach, we set out to dissect the genomic architecture underpinning these divergent predatory strategies. We reveal how the acquisition and refinement of distinct functional modules—from polysaccharide-degrading enzymes (CAZymes) to chemotaxis and quorum sensing systems—drive the evolution of specialist and generalist lifestyles, providing a high-resolution model of microbial adaptation in the marine environment.

## MATERIALS AND METHODS

### Algal cultures and algicidal bacteria

Algal cultures were grown at 20°C with a light intensity of 100 mol photons ms^−2^•s^−1^ and 14:10 h light:dark cycle. For dinoflagellate species, algal cultures were incubated with F/2-Si medium (lacking silicate). For diatom species, algal cultures were incubated with F/2+Si medium.

The *Alteromonas macleodii* strains FDHY-03 and FDHY-CJ were isolated from phytoplankton bloom-associated seawater samples collected from different coastal regions in Fujian Province, China. FDHY-CJ was isolated on 2 March 2017, from surface seawater during a diatom (*Skeletonema costatum*) bloom in the coastal area near Changle Airport, Fuzhou, Fujian. FDHY-03 was obtained on 24 May 2017, from surface seawater during a dinoflagellate (*Prorocentrum donghaiense*) bloom in Xiapu Bay, Ningde, Fujian. They were previously selected as algicidal bacteria (11, 12). The strains were stored at −80℃ (in 25% [vol/vol] glycerol).

### Co-cultivation of FDHY-CJ and FDHY-03 strains with different HABs

Bacterial cultures were co-cultured with 10 mL of algal cultures at a concentration of 2% (vol/vol) for 24 h. The control group was set up using the same amount of sterile culture medium. Set up three replicates for each algal strain treatment. After co-cultivation, the medium was fixed with Lugol’s iodine solution and stained for microscopic observation and counting of cell morphology. The algicidal rate was calculated using the following formula:


(1) $$Algicidal\;rate\;(\%)=\left(1-N_{1}/N_{0}\right)\times 100\%$$

where *N*_0_ represents the number of algal cells in the control group, and *N*_1_ represents the number of algal cells in the treatment group.

### Genomic DNA extraction, whole-genome sequencing, assembly

We employed a hybrid sequencing strategy combining Oxford Nanopore long-read and Illumina short-read technologies for the whole-genome assembly of strain FDHY-CJ. Genomic DNA was extracted using an optimized SDS method (13). Following purification, a 1D library was constructed for Nanopore sequencing using the SQK-LSK110 kit, while a short-insert library was prepared for Illumina sequencing using the VAHTS Universal Plus DNA Library Prep Kit. Sequencing was performed on the PromethION and NovaSeq 6000 platforms. After filtering out low-quality and short reads, the genome was assembled using Unicycler (version 0.5.0) (14) with long reads and subsequently polished using Illumina short reads with Pilon. All sequencing and assembly services were provided by Nanjing Aoweisen Gene Technology Co., Ltd.

For the whole-genome assembly of strain FDHY-03, we employed a hybrid sequencing strategy combining PacBio long-read and Illumina short-read technologies to ensure both continuity and base-level accuracy. Genomic DNA was extracted using the SDS method as described above. For long-read sequencing, a SMRTbell library was constructed using the SMRTbell Template Prep Kit and sequenced on the PacBio platform. For error correction and polishing, a standard paired-end library was prepared and sequenced on the Illumina HiSeq 2500 platform (PE150). The genome assembly was performed using a hierarchical approach. First, the PacBio long reads were assembled into contigs using Canu. Subsequently, the high-quality Illumina short reads were mapped to the assembled contigs to correct sequencing errors and improve consensus quality. All sequencing and assembly services were provided by Shanghai Majorbio Bio-pharm Technology Co., Ltd. The raw sequence data from this study have been deposited in the NGDC with accession numbers: CRA034289 and CRA034292.

### Gene prediction and functional annotation

CGViewer Server is also used to create circular genome maps in order to visualize sequence characterization information from sequence analysis results. Sequence comparisons are also performed in this server (15). Coding gene prediction was predicted using Prokka software (version: 1.14.6) for the assembled genome including CDS, tRNA, and rRNA subunits. MinCED (version: 0.4.2), islandpath (version: 1.0.6), PhiSpy (version: 4.2.21) were used to for gene structure prediction, including Clustered Regularly Interspaced Short Palindromic Repeats (CRISPRs), prophages, and genetic islands (GIs). Five databases were used to predict gene functions of FDHY-CJ. They were Gene Ontology (GO) (16), Clusters of Orthologous Group (COG) (17), the Kyoto Encyclopedia of Genes and Genomes (KEGG) (18), NCBI non-redundant protein (Nr) databases and Refseq.

In order to identify genes associated with algal lysing factors, all predicted gene and protein sequences were annotated through proprietary database, including Antibiotic Resistance Genes Database (ARDB) (19), Pathogen Host Interactions (PHI) (20), The Comprehensive Antibiotic Research Database (CARD) (21), Virulence Factors Database (VFDB) (22), and The Transporter Classification Database (TCDB) (23). Furthermore, the function of carbohydrate enzyme genes was annotated using the HMMER (24) based on the carbohydrate-active enzymes (CAZymes) (25) (*E*-value < 1*e*^−18^; coverage > 0.35).

### Phylogenetic Analysis

The phylogenomic tree was constructed by Type (Strain) Genome Server (TYGS) to phylogenetic identity of the strain FDHY-CJ and FDHY-03 (26). Average nucleotide identity (ANI) between strain FDHY-CJ, FDHY-03, and other *Alteromonas* strains was calculated using the orthoANI software (version 0.93.1) (27). The reference strains included *A. macleodii* ATCC 27126 (NC_018632.1), *A. mediterranea* DE (CP001103.3), *A. alba* 190 (NZ_PVNP01000003.1), *A. australica* H 17 (CP008849.1), *A. stellipolaris* R10SW13 (CP014322.1), *A. lipotrueae* MD_652 (NZ_JAESDI010000010.1), *A. genovensis* LMG 24078 (NZ_JAAAWO010000001.1), and *A. gliva* chi3 (NZ_JAQQXP010000001.1).

### Covariance and pan-genomic analysis

To further investigate evolutionary relationships among the species, we analyzed the genomic structure and synteny between strain FDHY-CJ and reference strains (FDHY-03, ATCC 27126, DE, R10SW13) using progressiveMauve alignment in Mauve software (version 20150226) (28). To investigate the diversity of the genus *Alteromonas*, Pan-genomic analysis of 10 strains of *Alteromonas* was performed using IPGA (29). PANOCT, OrthoMCL, Roary, panX, OrthoFinder, Panaroo, and PPanGGoLiN modules were selected for the analysis, and the parameters Identity, Ratio, and Support were set to 70, 0.95, and −1, respectively.

### Ocean Gene Atlas

#### Computation of unigene abundance/expression and taxonomic community composition

The eukaryote-enriched Marine Atlas of the Tara Ocean Unigenes (MATOU) (30) and the prokaryote-enriched Oceans Microbiome Reference Gene Catalog (OM-RGC.v2) (31) were queried in the Marine Gene Atlas (32). Unigene abundance/expression was calculated using RPKM (Reads Per Kilobase covered per Million of mapped reads) and standardized in two ways according to the analysis requirements. In biogeographic distribution, for OM-RGC abundance, the standardized method (average copy number per cell) was applied to each sample by dividing the total abundance of homologous genes by the median observed abundance of 10 prokaryotic single-marker genes. MATOU abundance is expressed as the coverage per gene per sample divided by the RPKM value (Reads Per Kilobase covered per Million of mapped reads). The output results include comparison results, homologous sequence FASTA files, standardized abundance of homologous sequences, and environmental data (*E*-value < 1*e*^−20^). Results were visualized using Tableau software (Version 2025.1) (33).

#### Spearman correlation analysis

We performed correlation analysis between gene expression/abundance in each taxonomic group and various environmental variables by calculating the Spearman correlation coefficient (Rho, *ρ*) using the corTest package in R (version 4.5.1). The results are presented in a heat map, with the significance level (*P* < 0.05) marked in the heat map. The complete correlation results are detailed in Supplementary Table (Table S5). The results were visualized using TBtools (version 2.326) (34).

## RESULTS AND DISCUSSION

### Differential algicidal activities of strains FDHY-03 and FDHY-CJ against algal species

While extensive strain-level diversity in algicidal activity is well-documented in its sister genus, *Pseudoalteromonas* (35) such functional variation within the *Alteromonas* genus itself has been far less explored. Although it is widely recognized that different species exhibit distinct capabilities, the genomic basis for the stark ecological differences between closely related strains within the same species remains poorly understood. Understanding this “ecological microdiversity” is, therefore, key to deciphering the mechanisms behind the cosmopolitan distribution of *Alteromonas* and the multifaceted roles its various strains play in marine ecosystems. As a classic copiotrophic bacterium, *Alteromonas* is known to play a significant role in regulating algal bloom dynamics (36, 37).

**Fig 1:**
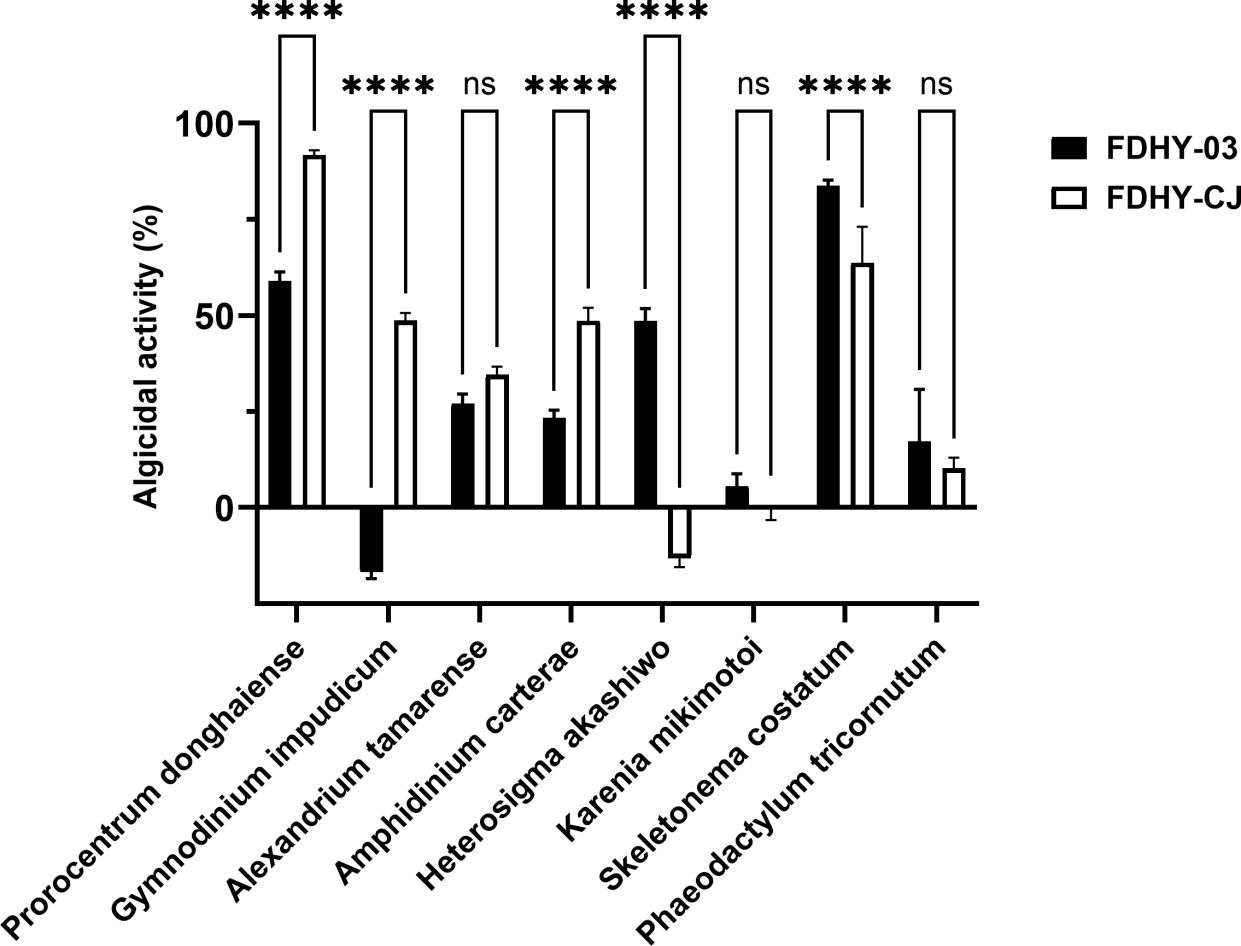
Algicidal bacteria FDHY-03 and FDHY-CJ dissolve different types of HABs. All error bars indicate the standard error of three replicates. (****, *P* < 0.0001; ns, *P* ≥ 0.05)

To compare the algicidal activity between strains FDHY-CJ and FDHY-03, algicidal tests were conducted against eight common harmful algal bloom species (Fig. 1). Significant differences in algicidal activity between the two strains were observed in six out of eight algal species. FDHY-03 demonstrated substantially higher algicidal activity against *Prorocentrum donghaiense*, *Gymnodinium impudicum*, *Alexandrium tamarense*, *Amphidinium carterae*, and *Heterosigma akashiwo*. Notably, FDHY-CJ showed no detectable activity against *G. impudicum* and *H. akashiwo*, while FDHY-03 reached about 50% activity. Against *Skeletonema costatum*, FDHY-CJ exhibited significantly higher activity than FDHY-03. No significant difference in algicidal activity was observed between the two strains for *Karenia mikimotoi* or *Phaeodactylum tricornutum*.

### Genome characteristics of *Alteromonas macleodii* FDHY-CJ and FDHY-03

The features of the genome of FDHY-CJ and FDHY-03 are shown in Table S1 and Fig S1. The complete genome of FDHY-CJ has a total length of 4,816,531 bp, with a GC content of 44.73%. The genome of strain FDHY-CJ is composed of a circular chromosome (4,575,219 bp), a plasmid (238,992 bp), and an unclassified fragment (2,320 bp). The number of 4,160 coding genes was predicted. There were 71 tRNAs, 16 rRNAs (5S + 16S + 23S), 3 CRISPR structures, 15 Prophages, and 8 genomic islands. Furthermore, there are 3,018 GO annotations, 3,142 KEGG annotations, 3,364 COG annotations, and 4,129 NR annotations.

The complete genome of FDHY-03 has a total length of 4,853,548 bp. It consists of one chromosome (4,744,891 bp) and a plasmid (108,657 bp). The G+C content of strain was 44.62%. A total of 4,172 coding genes including 71 tRNAs, 16 rRNAs (5S + 16S + 23S), and 11 genomic islands were predicted in the genome. Prophages and CRISPRs were absent in FDHY-03. The genome of strain FDHY-03 annotated 4,155 genes in the NR database and annotated in the COG database out of 3,329 genes, 2,948 genes were annotated in the GO database, and 2,364 genes were annotated in the KEGG database.

Specifically, there is a significant difference in the CRISPRs and prophage load between FDHY-CJ and FDHY-03. CRISPRs serve as an immune defense, while prophages drive horizontal gene transfer (HGT) (38, 39). The contrast suggests that FDHY-CJ, with its multiple CRISPRs and prophages, likely faced strong phage pressure, requiring robust defense mechanisms. In contrast, FDHY-03, lacking these elements, may have evolved in a low-phage environment or with different survival strategies (40). This divergence in defense and gene acquisition likely shaped their accessory genomes, with FDHY-CJ’s prophages possibly contributing specific genes, while FDHY-03 may rely more on plasmids or genomic islands for adaptive traits.

### Phylogenetic placement and genomic delineation of strains FDHY-CJ and FDHY-03

**Fig 2:**
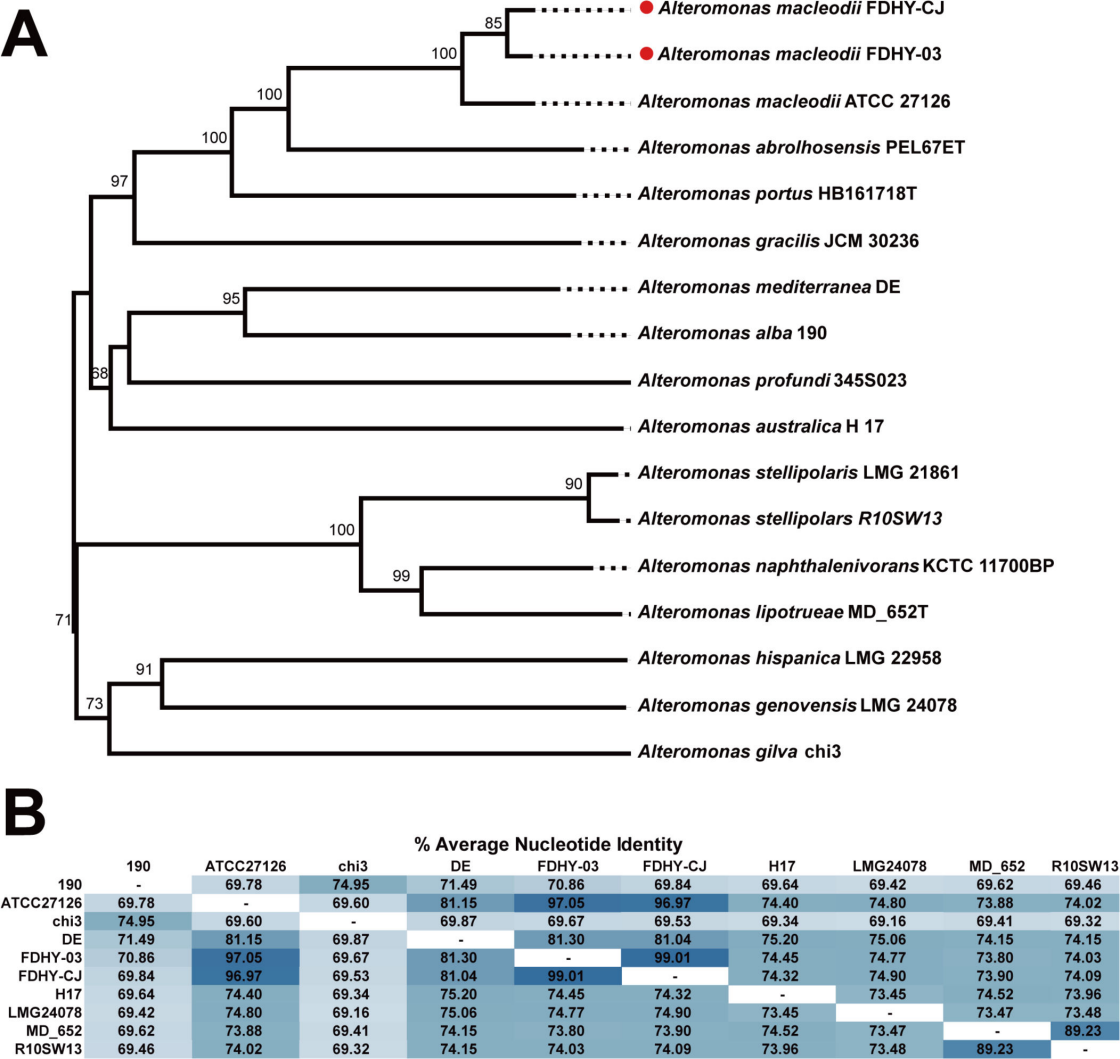
Phylogenetic tree construction and ANI value heat map. (**A**) Phylogenetic relationships between FDHY-CJ, FDHY-03, and other members of the genus *Alteromonas*. Phylogenomic tree constructed using Type (Strain) Genome Server depicting the position of strain FDHY-CJ, FDHY-03 among the genus of *Alteromonas*. The branch lengths are scaled in terms of GBDP distance formula d5. The numbers at the nodes are GBDP pseudo-bootstrap support values from 100 replications. (**B**) Heat map generated with OrthoANI, among closely related species of *Alteromonas* strain.

To determine the precise taxonomic identity of strains FDHY-CJ and FDHY-03, we conducted a comprehensive phylogenetic analysis combined with a genome-wide Average Nucleotide Identity (ANI) comparison (26, 27). The phylogenetic tree revealed that strains FDHY-CJ and FDHY-03 robustly clustered with the type strain *Alteromonas macleodii* ATCC 27,126, forming a distinct monophyletic clade with a high bootstrap support value of 100 (Fig. 2A). Other *Alteromonas* species, such as *A. alba* 190 and *A. gilva* chi3, occupied more distant branches, indicating a clear evolutionary separation. This phylogenetic placement was further corroborated by OrthoANI analysis (Fig. 2B), which revealed exceptionally high ANI values among the three *A. macleodii* strains, ranging from 96.97% to 99.01%. Notably, the ANI value between FDHY-CJ and FDHY-03 was 99.01%, indicating they are very closely related strains. All these values are well above the established 95%–96% threshold for species delineation, confirming they belong to the same species (41). In sharp contrast, the ANI values between this clade and other *Alteromonas* species were substantially lower (ranging from 69.34% to 89.23%), aligning perfectly with the branching pattern of the phylogenetic tree. Collectively, the congruent results from both the phylogenetic tree and the genome-wide ANI analysis unambiguously identify strains FDHY-CJ and FDHY-03 as *Alteromonas macleodii*. However, this remarkable genomic similarity stands in stark contrast to their divergent genomic architectures and ecological functions. This stark contrast-related strains underscores the extreme plasticity of the accessory genome in marine bacteria.

### Comparative genomics analysis

**Fig 3:**
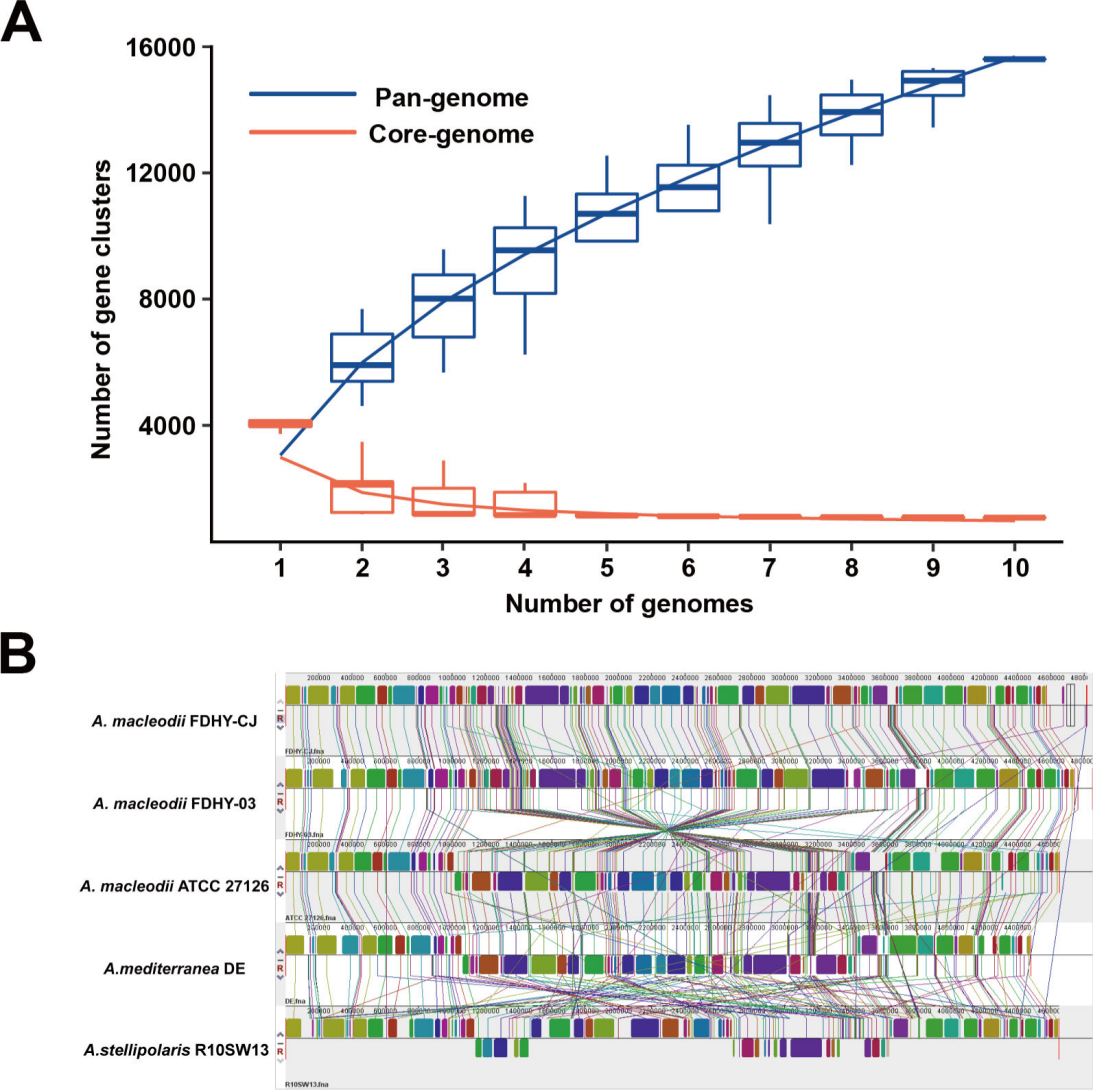
Comparative genomics analysis of *Alteromonas* species. (**A**) Core/pan-genome plot of *Alteromonas* species. (**B**) Genomic collinearity analysis of five strains of *Alteromonas*. Each row in the figure represents the linear genome of a strain, and the colored modules (Locally Collinear Blocks [LCBs]) indicate homologous regions conserved in different genomes. LCBs of the same color indicate gene regions with covariance across strains.

To understand the genomic diversity and evolutionary dynamics of the *Alteromonas* genus, we conducted a pan-genome analysis across 10 representative strains. The results reveal a highly dynamic and open pan-genome architecture. As additional genomes were incorporated, the total pan-genome continuously expanded to 6,042 gene families, while the core genome converged on a stable set of 3,225 genes (Fig. 3A). This trajectory is the hallmark of a genus with high genomic plasticity, indicating a remarkable capacity to acquire novel genetic material from the environment. This flexible accessory gene pool is a key evolutionary strategy, enabling *Alteromonas* to rapidly adapt to a wide array of marine ecological niches (42, 43).

This genomic plasticity is further evident at the level of chromosome structure. Synteny mapping among five complete genomes revealed extensive structural rearrangements—including inversions, translocations, and insertions—that reflect their evolutionary divergence (28). The degree of collinearity neatly mirrored the phylogenetic relationships. As expected, the two newly isolated strains, FDHY-CJ and FDHY-03, exhibit a high degree of structural conservation, with large, syntenic blocks reflecting their close kinship. The reference strain ATCC 27126 maintains substantial synteny with them but displays several local rearrangements, signaling incipient evolutionary divergence. In contrast, the more distantly related species, *A. mediterranea* DE and *A. stellipolaris* R10SW13, show a profound loss of collinearity, indicative of extensive genome shuffling over evolutionary time (Fig. 3B).

These structural variations, juxtaposed with the conserved core genome, highlight a key feature of *Alteromonas* evolution: a stable functional core is maintained while the genome’s architecture is actively reshaped. This structural plasticity is likely a primary mechanism driving adaptation to specific hosts and fluctuating marine environments.

### Carbohydrate-activated enzymes analysis

The initial attack on the algal cell wall is a critical step in bacterial algicidal processes. Given the vast diversity of polysaccharide structures in marine phytoplankton (e.g., cellulose, pectin, alginans), this process is orchestrated by a sophisticated and often species-specific repertoire of Carbohydrate-Active EnZymes (CAZymes) (44). Our previous work demonstrated that *Alteromonas* strains FDHY-03 and FDHY-CJ display distinct algicidal specificities (11, 12). This functional divergence prompted a comparative genomic analysis of their CAZyme arsenals to uncover the genetic basis for their different predatory behaviors, with the reference strain *A. macleodii* ATCC 27126 included for context.

**Fig 4:**
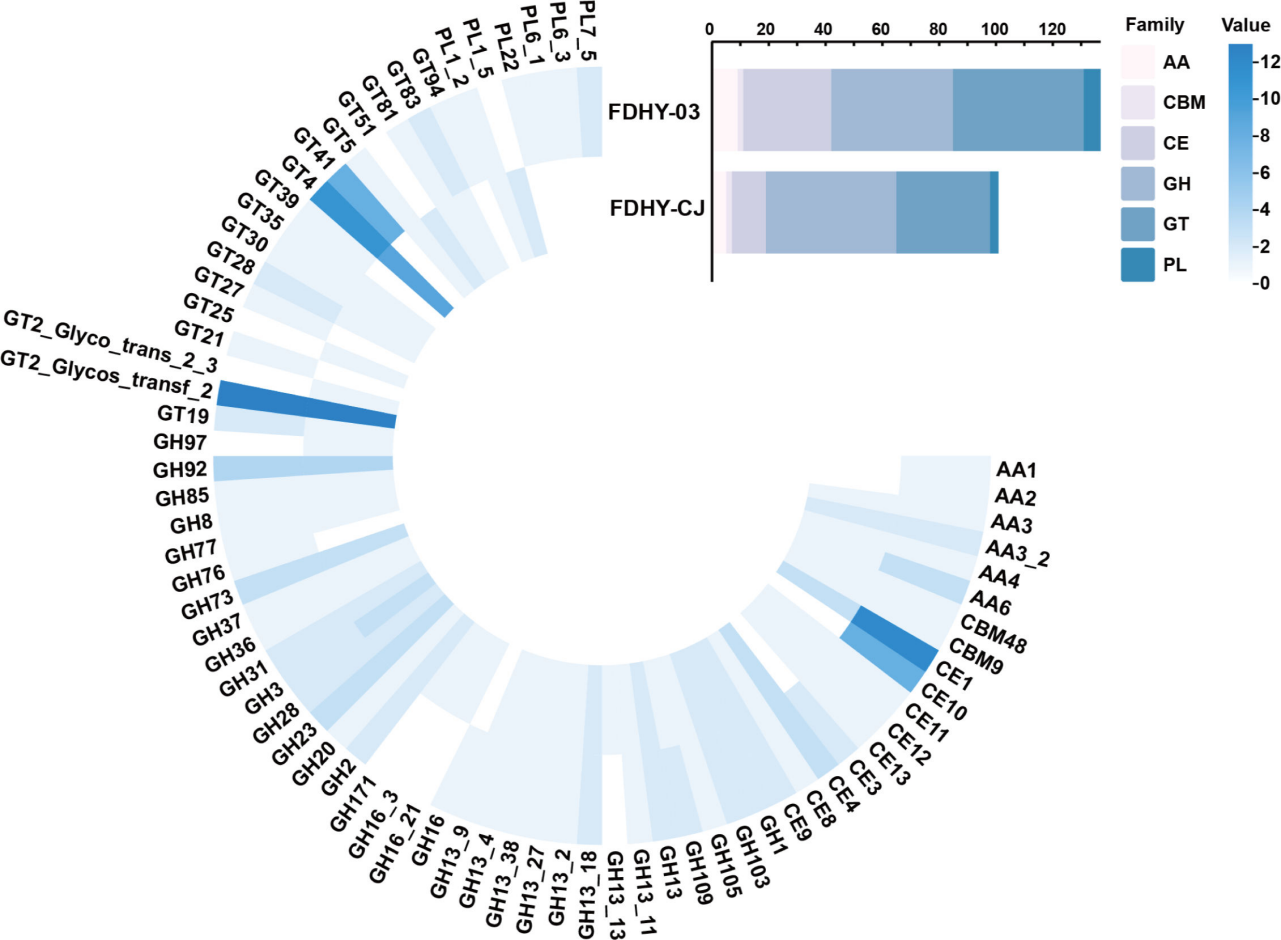
Carbohydrate-active enzymes (CAZymes) annotated in strain FDHY-CJ and FDHY-03.

**Fig 5:**
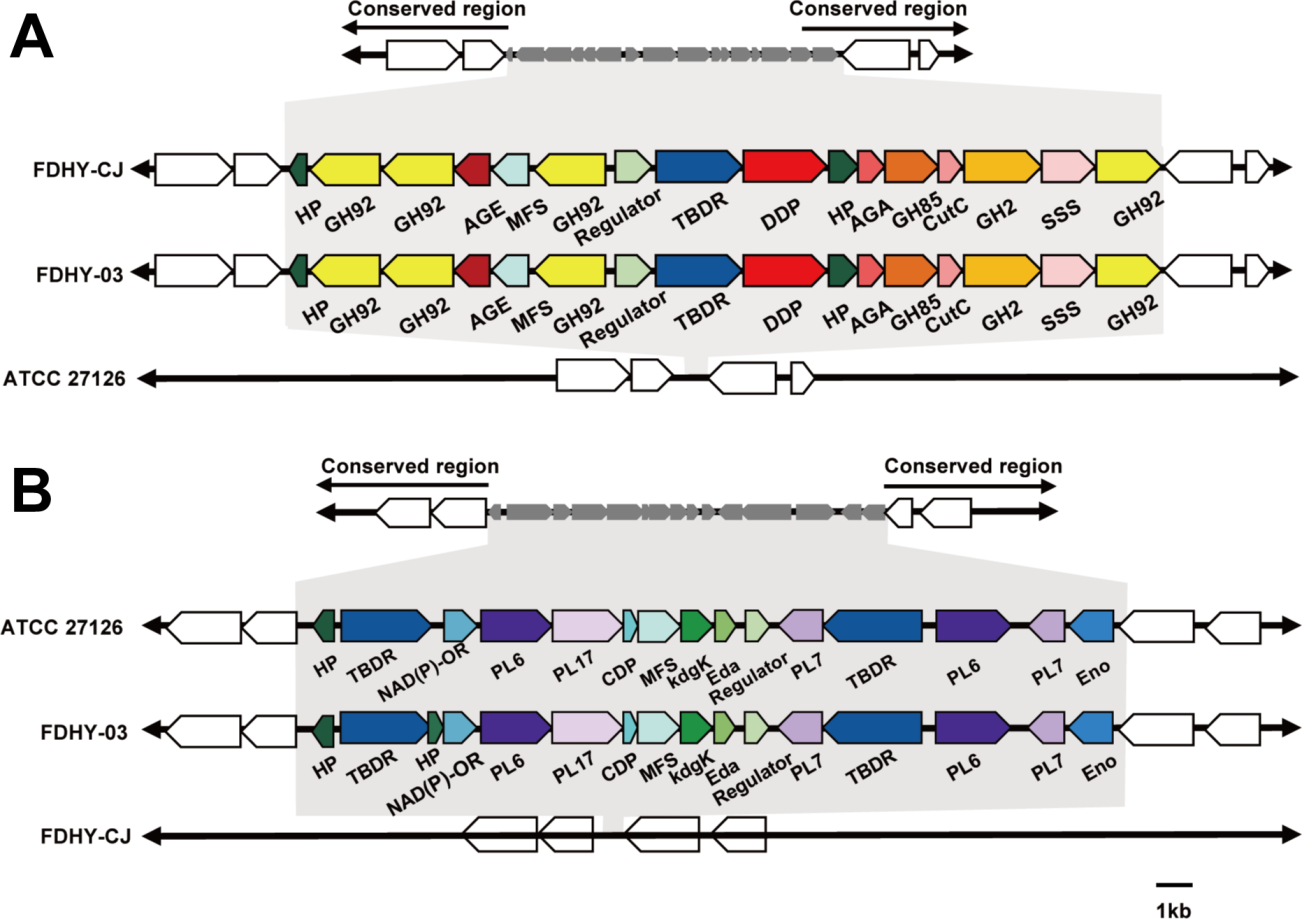
Comparative genomic analysis of carbohydrate-active enzymes and selected gene clusters in *Alteromonas macleodii* strains FDHY-CJ, FDHY-03, and ATCC 27126. (**A**) Gene cluster for glucuronomannan degradation. Key: TBDR, TonB-dependent receptor; MFS, major facilitator superfamily transporter; SSS, Na+:solute symporter; LacI, LacI family transcriptional regulator; AGA, N (4)-(β-N-acetylglucosaminyl)-L-asparaginase; HP, hypothetical protein. (**B**) Alginolytic system (AS) gene cluster. Core components include alginate lyases (PL6, PL17), an adjacent cupin-domain protein (CDP), a sugar permease (MFS), a sugar kinase (KdgK), and an isobutyrate dehydrogenase (Eda). Key: TBDR, TonB-dependent receptor; Eno, phosphopyruvate hydratase; HP, hypothetical protein.

A key genomic adaptation in both FDHY-03 and FDHY-CJ is the presence of inserted Polysaccharide Utilization Loci (PULs) enriched in glycoside hydrolases (GHs), including families GH92, GH85, and GH2 (Fig. 5A). This shared feature, absent in the reference strain, likely reflects their co-evolution with phytoplankton and provides a foundational capacity to degrade common algal polysaccharides. However, a closer inspection reveals a strategic divergence in their enzymatic toolkits. FDHY-CJ, the diatom specialist, uniquely encodes GH97 and a diverse array of GH16 subtypes (Fig. 4 and 5B). This specific enzymatic toolkit is precisely matched to the composition of the diatom *S. costatum* cell wall, which is fortified with structural polysaccharides like β-1,3-glucans (targeted by GH16). We propose that this specialized arsenal of hydrolases likely constitutes the primary defense mechanism by which FDHY-CJ efficiently dismantles diatoms, thereby explaining its potent and specific algicidal activity (95.45% lysis rate) against this target (12).

In contrast to the targeted hydrolase-centric strategy of FDHY-CJ, strain FDHY-03 employs a more versatile, synergistic enzymatic approach befitting a generalist predator. Its genome is distinguished by an expanded repertoire of polysaccharide lyases (PLs), including families PL22, PL6, and PL7_5, complemented by a rich inventory of auxiliary oxidoreductases (AAs), carbohydrate esterases (CEs), and glycosyl transferases (GTs) (Fig. 4 and 5B). This integrated enzymatic machinery allows for a multi-pronged attack on complex, decorated polysaccharides. Such synergistic degradation, where lyases, esterases, and hydrolases work in concert, has been shown to be crucial for breaking down recalcitrant algal polymers like ulvan and fucoidan in other marine bacteria (45, 46). Therefore, we speculate that this enzymatic versatility underpins the broader algicidal spectrum of FDHY-03, equipping it to successfully lyse a more diverse range of harmful algal bloom species.

In the algicidal assays, both FDHY-03 and FDHY-CJ exhibited no significant algicidal activity against two phylogenetically distinct algae species: *Karenia mikimotoi* and *Phaeodactylum tricornutum* (Fig. 4). This shared phenotype strongly suggests that the underlying cause is mechanistic rather than random. We hypothesize that the unique cell wall structures of these algae represent multiple defensive barriers: the silica-based shell of *P. tricornutum* and the amphiesma of *K. mikimotoi* (47, 48), all of which are resistant to the CAZyme repertoire carried by these two *Alteromonas* strains.

### Quorum sensing

Beyond their divergent enzymatic toolkits, strains FDHY-CJ and FDHY-03 exhibit fundamentally different Quorum Sensing (QS) architectures, suggesting distinct strategies for environmental perception and coordinating group behaviors. This divergence reflects two alternative evolutionary pathways for adapting to life in the dynamic algal phycosphere (49, 50). Strain FDHY-CJ employs a sophisticated and dual-mode QS strategy, which was equipped with a canonical AHL-based QS system, possessing a AHL synthase genes homolog for N-acyl homoserine lactone (AHL) synthesis, a common bacterial language for regulating processes like biofilm formation and enzyme secretion (49). More strikingly, its genome features an expanded solo-LuxR regulatory architecture, comprising seven putative LuxR-family transcriptional regulators that lack cognate LuxI synthase partners (Table S2). This configuration, also observed in other algae-associated bacteria like *Sulfitobacter* and *Phaeobacter*, is indicative of a specialized surveillance system (50). We hypothesize that these solo-LuxR proteins function as promiscuous receptors, enabling FDHY-CJ to intercept and interpret a wide range of chemical cues—from AHLs produced by competing bacteria to potential host-derived molecules—thereby allowing it to fine-tune its algicidal behavior in response to a complex chemical landscape. In stark contrast, and consistent with the majority of sequenced *A. macleodii* genomes, FDHY-03 lacks any identifiable AHL synthase genes and appears to have abandoned this mode of communication. Instead of producing specific signaling molecules, FDHY-03 likely perceives its population status through metabolic feedback and nutritional cues. Its genome uniquely harbors genes such as gabB (succinate-semialdehyde dehydrogenase) and livK (branched-chain amino acid ABC transporter), which are integral to central metabolism and nutrient sensing. The expression of such genes is often tightly linked to nutrient availability and cell density, providing an indirect but effective mechanism for coordinating population-level behaviors. This suggests an evolutionary trade-off: FDHY-03 has optimized a strategy based on sensing the general nutritional landscape, while FDHY-CJ has invested in a more complex and interactive system for specific intercellular and cross-kingdom communication.

Although the genus *Alteromonas* is cosmopolitan in marine environments (Fig. S2), the capacity for AHL synthesis appears to be a relatively uncommon trait within the population. An analysis of the Tara Oceans database showed that the AHL synthase gene was detected in 67.07% of metagenomic samples containing *Alteromonas* (Table S4). The AHL synthase gene in these samples may originate from other microorganisms besides *Alteromonas*. To estimate the prevalence of this gene within the *Alteromonas* population itself, we normalized its abundance against a genus-specific, single-copy marker the DUF6170 family protein gene (51). We scanned the genome sequences of the 10 strains mentioned above for collinearity analysis and found that this gene was a single copy in all 10 strains. Furthermore, this gene has no significant similarities in NCBI BLASTn when excluding *Alteromonodacea*. Thus, the DUF6170 family protein gene is an ideal biomarker for normalization. The normalized result revealed that the proportion of *Alteromonas* harboring the AHL synthase gene averaged 18.25%, ranging from 0.50% to 83.78% across samples (Table S4). Given that both the AHL synthase and DUF6170 family protein genes are single-copy in *Alteromonas*, this ratio serves as a reliable proxy for the fraction of the population capable of AHL production. Therefore, we conclude that the AHL-producing subpopulation constitutes a relatively small minority of the total *Alteromonas* community in the global ocean.

To investigate the ecological role of these specific strains, we analyzed their biogeography and *in situ* expression using the Ocean Genome Atlas (OGA). Metatranscriptomic data revealed that AHL synthase expression was primarily driven by *A. macleodii* in the 0.22–3 μm size fraction, with peak activity in the surface and deep chlorophyll maximum layers (Table S3). Geographically, the expression was highest in nearshore and equatorial/subtropical upwelling zones (Fig. S3). Crucially, the abundance of these transcripts correlated positively and significantly with high concentrations of key nutrients, including silicate (Si), nitrate (NO_3_^−^), and phosphate (PO_4_^3−^) (*P* < 0.05) (Table S5). This preference for nutrient-replete conditions aligns with the isolation source of our strain, FDHY-CJ, from a diatom bloom. The distinct positive correlation with silicate, an essential nutrient for diatoms, strongly implies that AHL-producing *A. macleodii* are selectively enriched in diatom-dominated marine environments. Thus, the possession and expression of the AHL synthase gene likely represent a key ecological strategy, enabling this specialized *A. macleodii* lineage to successfully colonize and thrive within the competitive, nutrient-rich niche created by diatom blooms.

### An expanded chemotaxis system may confer niche-specific algicidal activity

The algicidal activity of *Alteromonas* strains FDHY-CJ and FDHY-03 is primarily mediated by secreted extracellular metabolites rather than direct cell contact (11, 12). Effective delivery of these algicides and navigation within the competitive algal phycosphere necessitates a robust chemotaxis system. Genomic analysis confirmed that both strains possess a complete repertoire of genes for chemotaxis signaling (e.g., cheA/W/Y/B/R/D), flagellar motility, and multiple methyl-accepting chemotaxis proteins (MCPs) (Table S6 and S7), consistent with the adaptive strategies of copiotrophic surface-water bacteria. This machinery provides the fundamental basis for chemotactic behavior, enabling the bacteria to locate nutrient-rich microzones associated with algal cells (52).

While both strains are well-equipped for chemotaxis, a key distinction emerges in their sensory arsenal that may explain their different algicidal profiles: FDHY-CJ possesses an expanded and more specialized set of chemotaxis-related genes. Specifically, the FDHY-CJ genome uniquely encodes flgT and flgO, proteins known to enhance the structural integrity and efficiency of the flagellar motor, suggesting a capacity for more robust motility (53, 54).

Most notably, FDHY-CJ possesses the gene Tsr, which encodes a high-affinity chemoreceptor for the amino acid serine (55). This likely reveals the molecular mechanism involved in its specific binding to diatom hosts. Serine is not only a common component of dissolved organic matter (DOM) released by phytoplankton but, more importantly, as a structural amino acid, it exhibits specific enrichment in glycoproteins of diatom cell walls (56, 57). We, therefore, hypothesize that the Tsr receptor functions as a specific sensor, enabling FDHY-CJ to efficiently “home in” on chemical cues emanating from its preferred diatom algal targets, *S. costatum*. Thus, although both strains may share a core chemotaxis system, FDHY-CJ’s specialized sensory apparatus—composed of the Tsr receptor—enables it to recognize serine as a reliable “diatom marker.” This provides a critical competitive advantage, allowing it to bypass irrelevant signals in complex microbial environments and precisely locate and act upon specific algal targets.

### Conclusions

This study dissects the genomic underpinnings of profound functional divergence between two closely related strains of *Alteromonas macleodii*, revealing how specific variations in the accessory genome are consistent with the evolution of distinct predatory lifestyles. We present a clear divergence in their algicidal strategies—one defined by broad-spectrum versatility and the other by high-potency, target-focused efficacy—orchestrated by suites of functionally integrated genomic traits. Strain FDHY-03 employs a broad-spectrum approach, leveraging a versatile and synergistic enzymatic arsenal rich in polysaccharide lyases and esterases. This allows it to deconstruct a wide variety of algal cell walls, a strategy complemented by an environmental sensing system reliant on the sensing of broad metabolic cues. In contrast, strain FDHY-CJ has evolved an integrated, high-precision system highly adapted for targeting diatoms. This includes (i) an advanced targeting system, using specialized chemoreceptors like Tsr to home in on its algal targets; (ii) a tailored enzymatic toolkit, rich in β-glucanases (GH16) well-suited for degrading key components of the diatom cell wall; and (iii) a sophisticated communication and surveillance network, using AHL signals and solo-LuxR proteins to coordinate its behavior in the competitive, diatom-rich niches where we have shown this system is most active. Our findings provide a high-resolution snapshot of microbial microevolution, demonstrating how the open pan-genome and structural plasticity of *Alteromonas* facilitate the rapid acquisition and refinement of functional modules for niche partitioning. This work not only illuminates the intricate molecular dialogs that structure marine microbial communities but also underscores a critical principle for applied science: the effective use of bacteria for controlling harmful algal blooms depends on a deep, genomic-level understanding of their predatory mechanisms. Moving forward, this “genome-to-phenotype” approach provides a powerful framework for discovering and deploying targeted microbial formulations to address specific ecological challenges. Future research will focus on experimentally verifying the causal relationship between these genes and the specific algicidal phenotype. Through experimental techniques such as enzyme activity assays, chemotaxis experiments, AHL addition, or knockout experiments, we can further validate the roles of genes including GH16, the Tsr receptor, and AHL synthase in regulating the algicidal phenotype.

## SUPPLEMENTAL MATERIAL

Supplemental material aem.01965-25-s0001.pdf Fig. S1 to S3 and Tables S1 to S7.

ASM does not own the copyrights to Supplemental Material that may be linked to, or accessed through, an article. The authors have granted ASM a non-exclusive, world-wide license to publish the Supplemental Material files. Please contact the corresponding author directly for reuse.
