# Supplementary material for: Comparative genomics reveals the molecular basis for divergent algicidal strategies in two Alteromonas macleodii strains
Source: Appl Environ Microbiol. 2025 Dec 29;92(1):e01965-25. doi: 10.1128/aem.01965-25 (PMC12838423; doi:10.1128/aem.01965-25)
Supplement: Supplemental material — Fig. S1 to S3 and Tables S1 to S7. [file aem.01965-25-s0001.pdf]

SUPPLEMENTARY MATERIAL

Comparative Genomics Reveals the Molecular Basis for Divergent Algicidal Strategies in Two *Alteromonas macleodii* Strains

Yuxin Lai[1,2], Xinyu Liu[1,4], Zhiyuan Chen[3], Yue Li[1,4], Xinguo Shi[1,4*]

1 Technical Innovation Service Platform for High Value and High Quality Utilization of Marine Organism, Fuzhou University, Fuzhou 350108, China;

2 College of Advanced Manufacturing, Fuzhou University, Jinjiang 362200, China;

3 Maynooth International Engineering College, Fuzhou University, Fuzhou 350108, China;

4 College of Biological Science and Engineering, Fuzhou University, Fuzhou, China

***Corresponding authors**

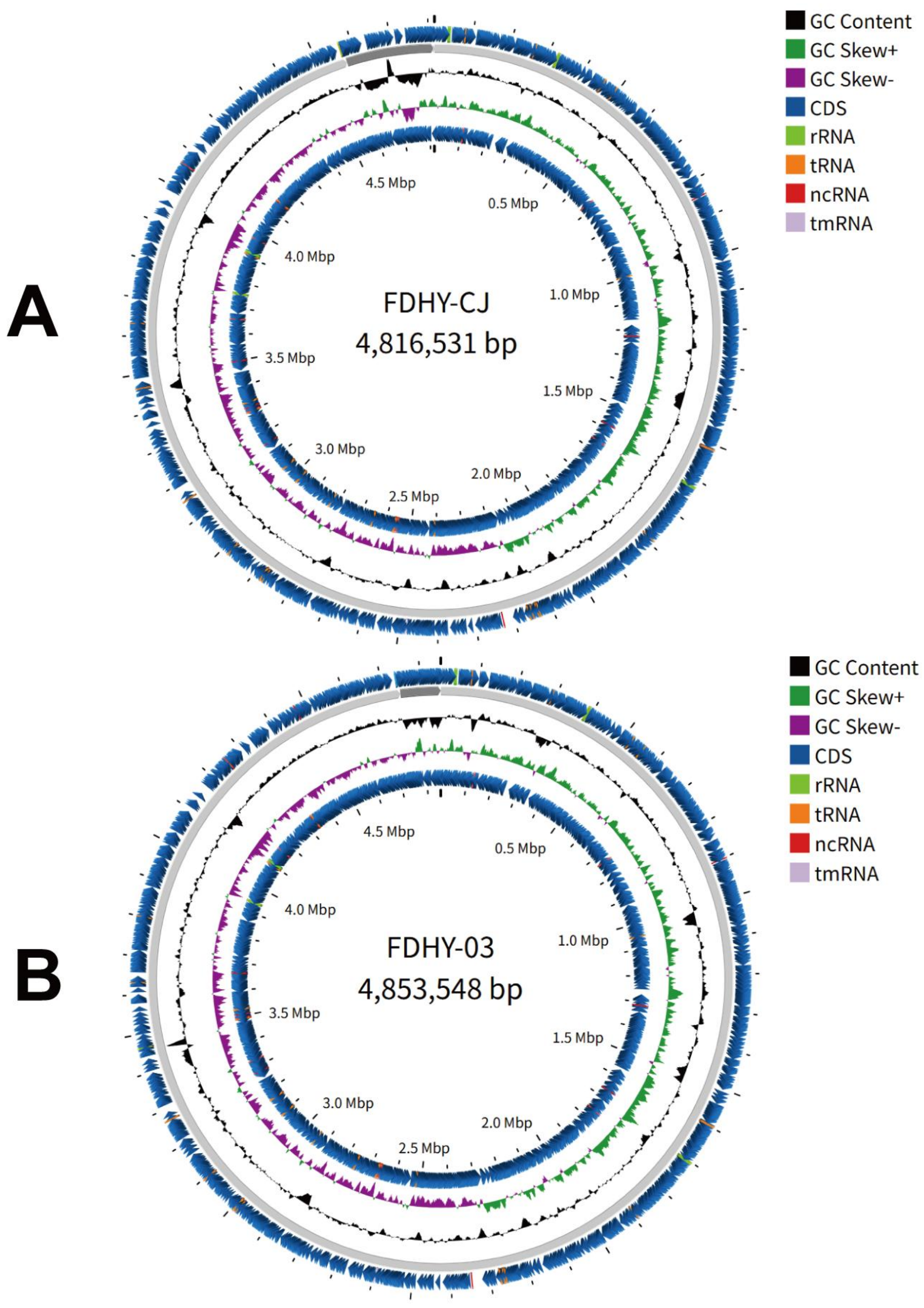

Figure S1 Circular genome maps of *Alteromonas macleodii* strains. (A) FDHY-CJ. **(B)** FDHY-03. The maps were generated using the CGView Server with default parameters.

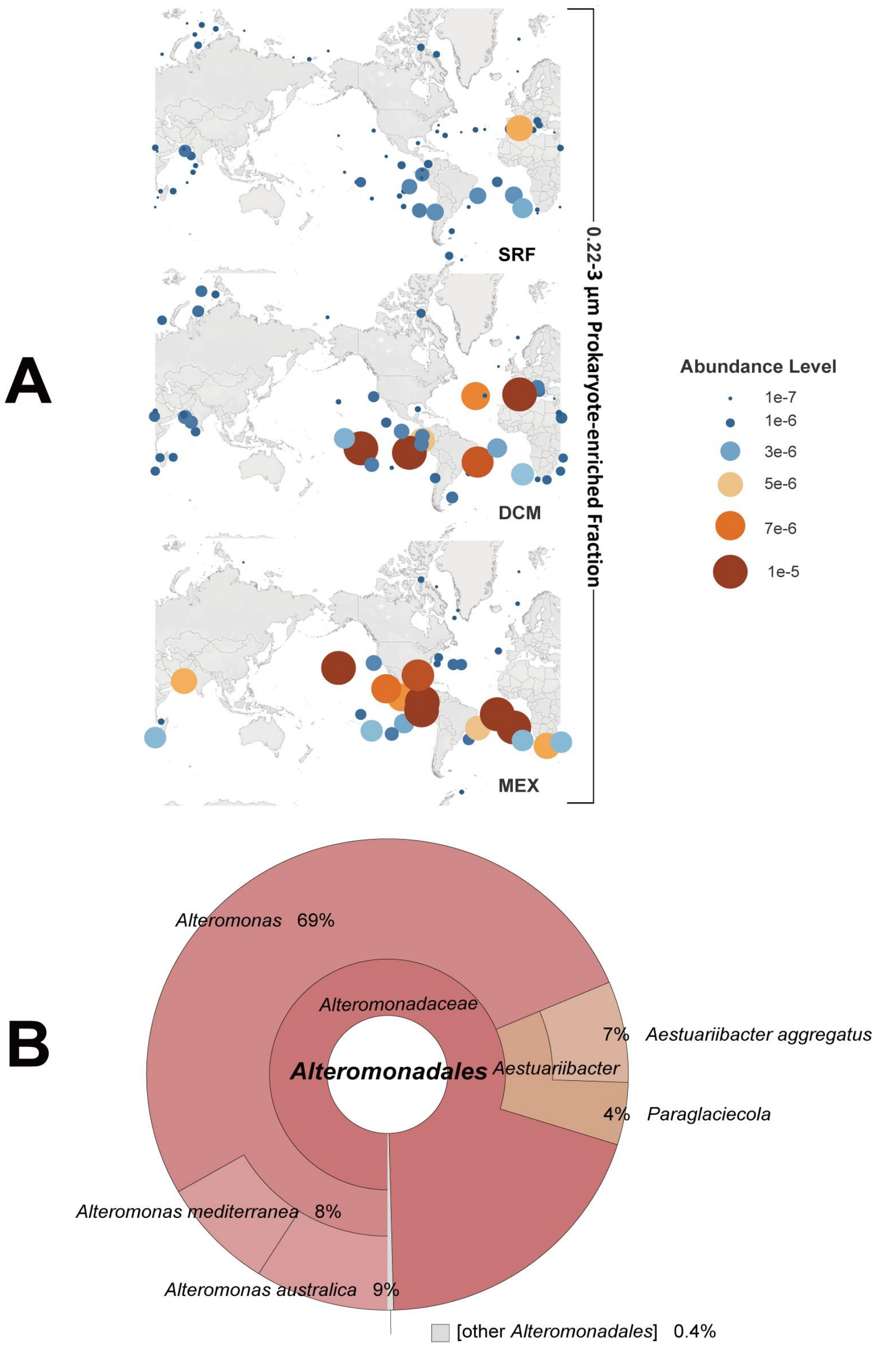

Figure S2 Tara Ocean Gene Atlas maps the prevalence and taxonomic distribution of *Alteromonas* across different marine regions and particle size fractions. (A) global ocean abundance across siza fractions, abundance Level represent percent abundance of MAGs containing hit genes in metagenomes of each size fraction. **(B)** Taxonomic distribution of hits.

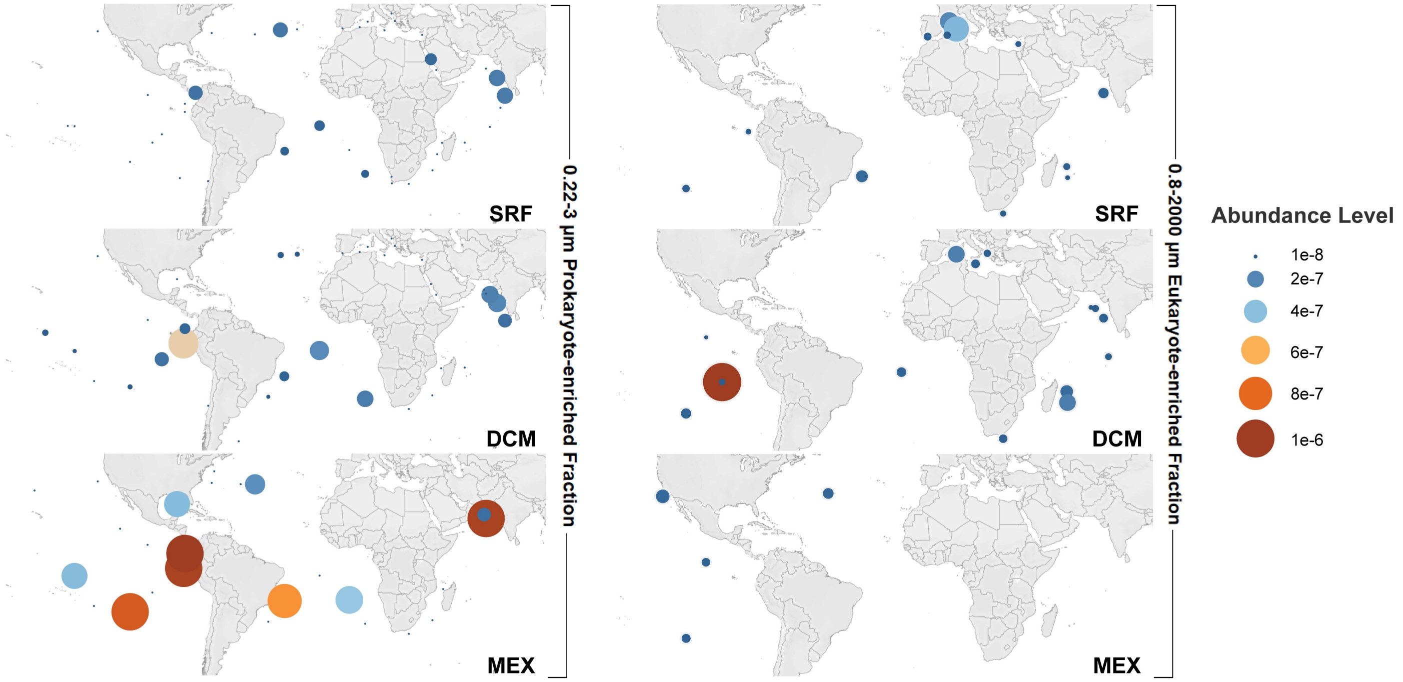

Figure S3 Distribution of AHL synthase genes in global oceans (a-f) in eukaryotic-enriched (0.8-2000**µ**m fraction) and prokaryotic-enriched (0.2-3**µ**m fraction) metatranscriptomes.

Table S1 General features of genomes.

	Strain	
	A. macleodii FDHY-CJ	*A. macleodii* FDHY-03
Genome size (bp)	4,816,531	4,853,548
GC content (%)	44.73	44.62
Predicted CDS	4160	4172
tRNA	71	71
rRNA	16	16
Plasmid	1	1
CRISPRs	3	0
Prophages	15	0
Genomic islands	8	11
Genes assigned to GOs	3018	2948
Genes assigned to KEGGs	3142	2364
Genes assigned to COGs	3364	3329
Genes assigned to NRs	4129	4155

Table S2 Quorum sensing-related genes of FDHY-CJ.

Gene ID	Gene name	Length	Description
2024	PvdQ	840	Acyl-homoserine lactone acylase PvdQ
3422	DegU	214	Bacterial regulatory proteins,luxR family
3433	NreC	209	Bacterial regulatory proteins,luxR family
3559	MalT	103	Bacterial regulatory proteins,luxR family
3259	DevR/DosR	219	Two component transcriptional regulator, LuxR family
3264	ExaE	225	Bacterial regulatory proteins,luxR family
3126	DesR	206	Bacterial regulatory proteins,luxR family
2578	DegU	222	Two component transcriptional regulator, LuxR family

Table S3 Statistics of sampling station and sample number in different water layers.

	SRF	DCM	MES	MIX	FSW	ZZZ	Total sampling stations
cvil metaT (OMRGC.v2)	21	21	15	1	0	1	59
cvil metaG (OMRGC.v2)	42	38	27	3	0	0	110
cvil metaT (MATOU)	14	21	5	0	2	0	42
cvil metaG (MATOU)	97	63	7	5	1	4	177
Alteromonas metaG (OMRGC.v2)	71	51	36	5	0	1	164

SRF:surfacelayer;
DCM:deepchlorophyllmaximumlayer;
MES:mesopelagiczone;
MIX:marineepipelagicmixedlayer;
FSW:filteredseawater;
ZZZ:marinewaterlayer.

Table S4 Spearman's correlations between environmental parameters and cvil gene expression level (metaT) or gene abundance (metaG). Spearman's correlation coefficients were denoted under given color scheme. Significant p-value ($p<0.05$) were labelled with blue. n=the number of the scatters.

(a) metaT (0.8-2000µm)

	coefficients (rho)	p-value	n
Seafloor (m)	-0.190	0.253	38
Temperature (°C)	-0.171	0.291	40
Salinity (PSU)	-0.153	0.346	40
Density (kg/m**3)	0.102	0.530	40
Distance_coast (km)	0.151	0.366	38
Chlorophyll_A (mg/m**3)	-0.210	0.193	40
Depth (m)	0.296	0.057	42
PAR (mol quanta/m**2/day)	-0.172	0.314	36
O2 (µmol/kg)	-0.341	0.045	35
NO3 (µmol/l)	0.112	0.569	28
Iron_5m* (µmol/l)	-0.044	0.784	42
Ammonium_5m* (µmol/l)	0.142	0.368	42
Nitrite_5m* (µmol/l)	0.050	0.753	42
Nitrate_5m* (µmol/l)	0.036	0.819	42
CDOM* (ppb (QSE))	-0.638	0.047	10
NPP_C* (mg/m**2/day)	-0.302	0.093	32
POC* (µg/kg)	-0.104	0.528	39
PIC* (mol/m3)	-0.113	0.523	34
Alkalinity (µmol/kg)	-0.153	0.418	30
Carbon Total (µmol/kg)	-0.429	0.018	30
CO2 (µmol/kg)	-0.564	0.322	5
CO3 (µmol/kg)	-0.311	0.094	30
HCO3 (µmol/kg)	0.108	0.571	30
pH (-)	0.564	0.322	5
NO2 (µmol/l)	-0.162	0.393	30
PO4 (µmol/l)	0.147	0.465	27
NO3_NO2 (µmol/l)	-0.050	0.794	30
Si (µmol/l)	0.110	0.568	29
MLD (m)	-0.013	0.941	36
DCM (m)	0.042	0.802	38
Depth_max_Brunt_Väisälä_freq (m)	-0.194	0.250	37
Depth_max_O2 (m)	-0.084	0.643	33
Depth_min_O2 (m)	-0.072	0.692	33
Depth_nitracline (m)	-0.007	0.972	25
Shannon_Darwin* (-)	0.205	0.246	34
Shannon_Physat* (-)	0.128	0.449	37
Chlorophyll c3 (mg/m**3)	-0.336	0.069	30
Peridinin (mg/m**3)	-0.257	0.170	30
Fucoxanthin (mg/m**3)	-0.429	0.018	30
Prasinoxanthin (mg/m**3)	-0.017	0.929	30
19-Hexanoyloxyfucoxanthin (mg/m**3)	-0.315	0.090	30
Alloxanthin (mg/m**3)	0.078	0.681	30
Zeaxanthin (mg/m**3)	-0.403	0.027	30
Lutein (mg/m**3)	-0.424	0.019	30
Latitude (-)	0.148	0.349	42
Longitude (-)	-0.157	0.322	42

(b) metaT (0.22-3µm)

	coefficients (rho)	p-value	n
Seafloor (m)	0.038	0.777	58
Temperature (°C)	-0.325	0.013	58
Salinity (PSU)	-0.164	0.217	58
Density (kg/m**3)	0.358	0.006	58
Distance_coast (km)	-0.028	0.837	58
Chlorophyll_A (mg/m**3)	-0.423	0.001	58
Depth (m)	0.296	0.023	59
PAR (mol quanta/m**2/day)	-0.320	0.032	45
O2 (µmol/kg)	-0.477	0.000	58
NO3 (µmol/l)	0.399	0.003	54
Iron_5m* (µmol/l)	0.122	0.356	59
Ammonium_5m* (µmol/l)	-0.056	0.676	59
Nitrite_5m* (µmol/l)	0.038	0.776	59
Nitrate_5m* (µmol/l)	-0.093	0.482	59
CDOM* (ppb (QSE))	-0.165	0.589	13
NPP_C* (mg/m**2/day)	0.177	0.294	37
POC* (µg/kg)	-0.001	0.992	49
PIC* (mol/m3)	-0.010	0.949	44
Alkalinity (µmol/kg)	-0.264	0.083	44
Carbon Total (µmol/kg)	-0.289	0.057	44
CO2 (µmol/kg)	-0.248	0.489	10
CO3 (µmol/kg)	-0.357	0.017	44
HCO3 (µmol/kg)	0.119	0.441	44
pH (-)	-0.285	0.425	10
NO2 (µmol/l)	-0.212	0.140	50
PO4 (µmol/l)	0.354	0.011	51
NO3_NO2 (µmol/l)	0.224	0.118	50
Si (µmol/l)	0.425	0.002	50
MLD (m)	-0.150	0.266	57
DCM (m)	-0.114	0.396	58
Depth_max_Brunt_Väisälä_freq (m)	-0.013	0.921	58
Depth_max_O2 (m)	-0.185	0.164	58
Depth_min_O2 (m)	0.163	0.220	58
Depth_nitracline (m)	-0.052	0.720	50
Shannon_Darwin* (-)	-0.061	0.672	51
Shannon_Physat* (-)	-0.196	0.169	51
Chlorophyll c3 (mg/m**3)	-0.317	0.036	44
Peridinin (mg/m**3)	-0.339	0.024	44
Fucoxanthin (mg/m**3)	-0.289	0.057	44
Prasinoxanthin (mg/m**3)	-0.078	0.614	44
19-Hexanoyloxyfucoxanthin (mg/m**3)	-0.322	0.033	44
Alloxanthin (mg/m**3)	-0.259	0.090	44
Zeaxanthin (mg/m**3)	-0.182	0.238	44
Lutein (mg/m**3)	-0.004	0.979	44
Latitude (-)	0.073	0.580	59
Longitude (-)	0.091	0.492	59

(c) metaG (0.8-2000µm)

	coefficients (rho)	p-value	n
Seafloor (m)	-0.022	0.772	173
Temperature (°C)	0.219	0.004	174
Salinity (PSU)	-0.032	0.671	174
Density (kg/m**3)	-0.162	0.033	174
Distance_coast (km)	0.088	0.248	173
Chlorophyll_A (mg/m**3)	-0.082	0.281	174
Depth (m)	0.123	0.103	177
PAR (mol quanta/m**2/day)	0.162	0.050	148
O2 (µmol/kg)	-0.379	0.000	159
NO3 (µmol/l)	0.006	0.941	135
Iron_5m* (µmol/l)	0.083	0.273	177
Ammonium_5m* (µmol/l)	0.048	0.522	177
Nitrite_5m* (µmol/l)	-0.176	0.019	177
Nitrate_5m* (µmol/l)	-0.289	0.000	177
CDOM* (ppb (QSE))	-0.101	0.471	53
NPP_C* (mg/m**2/day)	-0.181	0.035	136
POC* (µg/kg)	-0.159	0.052	150
PIC* (mol/m3)	-0.049	0.555	145
Alkalinity (µmol/kg)	-0.093	0.272	141
Carbon Total (µmol/kg)	-0.281	0.001	141
CO2 (µmol/kg)	-0.001	0.994	56
CO3 (µmol/kg)	-0.212	0.012	141
HCO3 (µmol/kg)	-0.120	0.155	141
pH (-)	-0.072	0.596	56
NO2 (µmol/l)	-0.166	0.037	158
PO4 (µmol/l)	0.090	0.263	155
NO3_NO2 (µmol/l)	-0.030	0.712	156
Si (µmol/l)	0.109	0.176	156
MLD (m)	-0.226	0.003	171
DCM (m)	0.091	0.233	173
Depth_max_Brunt_Väisälä_freq (m)	-0.054	0.485	171
Depth_max_O2 (m)	0.072	0.368	158
Depth_min_O2 (m)	0.097	0.225	158
Depth_nitracline (m)	-0.117	0.197	124
Shannon_Darwin* (-)	-0.054	0.510	150
Shannon_Physat* (-)	-0.095	0.228	161
Chlorophyll c3 (mg/m**3)	-0.224	0.008	141
Peridinin (mg/m**3)	-0.191	0.023	141
Fucoxanthin (mg/m**3)	-0.281	0.001	141
Prasinoxanthin (mg/m**3)	-0.123	0.148	141
19-Hexanoyloxyfucoxanthin (mg/m**3)	-0.206	0.014	141
Alloxanthin (mg/m**3)	-0.298	0.000	141
Zeaxanthin (mg/m**3)	0.024	0.775	141
Lutein (mg/m**3)	-0.129	0.126	141
Latitude (-)	-0.173	0.022	177
Longitude (-)	0.284	0.000	177

(d) metaG (0.22-3µm)

	coefficients (rho)	p-value	n
Seafloor (m)	-0.209	0.777	58
Temperature (°C)	-0.271	0.013	58
Salinity (PSU)	-0.071	0.217	58
Density (kg/m**3)	0.233	0.006	58
Distance_coast (km)	0.178	0.837	58
Chlorophyll_A (mg/m**3)	-0.015	0.001	58
Depth (m)	0.440	0.023	59
PAR (mol quanta/m**2/day)	-0.398	0.032	45
O2 (µmol/kg)	-0.235	0.000	58
NO3 (µmol/l)	0.306	0.003	54
Iron_5m* (µmol/l)	0.114	0.356	59
Ammonium_5m* (µmol/l)	0.146	0.676	59
Nitrite_5m* (µmol/l)	-0.039	0.776	59
Nitrate_5m* (µmol/l)	-0.162	0.482	59
CDOM* (ppb (QSE))	0.266	0.589	13
NPP_C* (mg/m**2/day)	0.064	0.294	37
POC* (µg/kg)	0.044	0.992	49
PIC* (mol/m3)	0.151	0.949	44
Alkalinity (µmol/kg)	-0.058	0.083	44
Carbon Total (µmol/kg)	-0.125	0.057	44
CO2 (µmol/kg)	0.157	0.489	10
CO3 (µmol/kg)	-0.088	0.017	44
HCO3 (µmol/kg)	0.054	0.441	44
pH (-)	0.004	0.425	10
NO2 (µmol/l)	0.130	0.140	50
PO4 (µmol/l)	0.230	0.011	51
NO3_NO2 (µmol/l)	0.244	0.118	50
Si (µmol/l)	0.228	0.002	50
MLD (m)	-0.103	0.266	57
DCM (m)	-0.006	0.396	58
Depth_max_Brunt_Väisälä_freq (m)	-0.073	0.921	58
Depth_max_O2 (m)	0.015	0.164	58
Depth_min_O2 (m)	0.015	0.220	58
Depth_nitracline (m)	-0.134	0.720	50
Shannon_Darwin* (-)	0.053	0.672	51
Shannon_Physat* (-)	0.238	0.169	51
Chlorophyll c3 (mg/m**3)	-0.108	0.036	44
Peridinin (mg/m**3)	-0.136	0.024	44
Fucoxanthin (mg/m**3)	-0.125	0.057	44
Prasinoxanthin (mg/m**3)	0.076	0.614	44
19-Hexanoyloxyfucoxanthin (mg/m**3)	-0.122	0.033	44
Alloxanthin (mg/m**3)	-0.163	0.090	44
Zeaxanthin (mg/m**3)	-0.172	0.238	44
Lutein (mg/m**3)	-0.299	0.979	44
Latitude (-)	-0.147	0.580	59
Longitude (-)	0.214	0.492	59

Table S5 TARA ocean AHL synthase abundance.

Sampling depth	Sample	AHL_synthas abundance (percent of total genes per sample)	DUF6170 abundance (percent of total genes per sample)	AHL_synthas abundance normalized to DUF6170
DCM	TARA_A100001011	1.05E-08	1.67E-08	62.76%
DCM	TARA_A100001037	9.00E-08	3.36E-07	26.83%
DCM	TARA_A200000159	1.12E-05	2.07E-05	54.16%
DCM	TARA_B000000441	8.55E-08	7.41E-07	11.53%
DCM	TARA_B000000557	1.74E-07	7.01E-07	24.83%
DCM	TARA_B100000029	2.41E-07	9.94E-07	24.24%
DCM	TARA_B100000035	1.43E-07	5.24E-07	27.29%
DCM	TARA_B100000073	4.67E-07	1.69E-06	27.65%
DCM	TARA_B100000085	4.18E-08	8.58E-08	48.74%
DCM	TARA_B100000131	2.55E-07	7.46E-07	34.19%
DCM	TARA_B100000214	9.91E-08	6.55E-07	15.13%
DCM	TARA_B100000287	1.23E-07	1.49E-06	8.27%
DCM	TARA_B100000405	2.55E-07	7.19E-07	35.45%
DCM	TARA_B100000427	9.84E-07	3.26E-06	30.19%
DCM	TARA_B100000482	6.94E-07	4.39E-06	15.80%
DCM	TARA_B100000519	1.80E-06	8.47E-06	21.25%
DCM	TARA_B100000530	1.90E-07	3.16E-07	60.09%
DCM	TARA_B100000614	1.86E-08	3.69E-06	0.50%
DCM	TARA_B100000700	5.77E-07	1.01E-05	5.74%
DCM	TARA_B100000767	7.69E-07	1.20E-06	63.88%
DCM	TARA_B100000902	1.91E-07	1.78E-06	10.71%
DCM	TARA_B100000927	1.39E-07	1.78E-06	7.81%
DCM	TARA_B100000945	1.34E-08	1.78E-06	0.75%
DCM	TARA_B100000959	1.49E-07	3.96E-06	3.76%
DCM	TARA_B100000965	2.58E-06	1.01E-05	25.49%
DCM	TARA_B100001029	2.89E-08	1.51E-07	19.17%
DCM	TARA_B100001059	2.80E-07	9.13E-07	30.66%
DCM	TARA_B100001094	9.78E-09	1.05E-06	0.93%
DCM	TARA_B100001113	6.23E-08	5.36E-06	1.16%
DCM	TARA_B100001559	1.32E-08	1.70E-07	7.76%
DCM	TARA_B100001778	3.70E-08	6.82E-06	0.54%
DCM	TARA_B100001996	9.10E-08	2.03E-06	4.49%
DCM	TARA_B100002052	3.88E-08	3.09E-07	12.57%
DCM	TARA_E500000081	3.29E-07	1.74E-06	18.94%
DCM	TARA_E500000331	1.81E-07	1.15E-06	15.69%
DCM	TARA_S200000501	1.32E-07	3.70E-07	35.64%
DCM	TARA_X000000368	1.48E-07	4.59E-07	32.21%
DCM	TARA_X000001036	2.84E-07	7.89E-07	35.99%
MES	TARA_B000000460	5.74E-07	5.94E-06	9.66%
MES	TARA_B100000097	3.28E-07	1.59E-06	20.69%
MES	TARA_B100000315	1.16E-06	5.77E-06	20.12%
MES	TARA_B100000378	5.22E-08	3.39E-07	15.40%
MES	TARA_B100000408	7.16E-07	4.02E-06	17.82%
MES	TARA_B100000446	1.98E-06	4.96E-05	3.99%
MES	TARA_B100000470	1.03E-06	3.96E-06	25.99%
MES	TARA_B100000508	1.28E-06	1.34E-05	9.54%
MES	TARA_B100000585	1.37E-07	3.40E-06	4.03%
MES	TARA_B100000678	1.04E-08	1.02E-06	1.02%
MES	TARA_B100000745	4.02E-07	1.17E-06	34.30%
MES	TARA_B100000749	1.23E-06	5.32E-06	23.14%
MES	TARA_B100000929	1.37E-06	1.71E-05	7.99%
MES	TARA_B100000949	9.61E-08	4.08E-06	2.35%
MES	TARA_B100000953	1.20E-06	1.03E-05	11.70%
MES	TARA_B100001013	1.83E-07	1.61E-06	11.38%

MES	TARA_B100001079	1.12E-07	1.97E-06	5.70%
MES	TARA_B100001105	7.98E-08	2.15E-06	3.72%
MES	TARA_B100001146	2.62E-08	6.56E-07	3.99%
MES	TARA_B100001167	9.51E-09	4.13E-07	2.30%
MES	TARA_B100001245	4.61E-07	1.12E-05	4.13%
MES	TARA_B100001750	7.56E-08	1.10E-06	6.86%
MES	TARA_B100001765	9.38E-08	1.10E-06	8.51%
MES	TARA_B100001971	1.05E-07	7.43E-06	1.41%
MES	TARA_B100002003	8.09E-08	6.58E-06	1.23%
MES	TARA_B100002049	3.86E-07	8.79E-06	4.39%
MES	TARA_Y100000294	2.14E-07	1.88E-06	11.39%
MIX	TARA_B100000676	4.26E-08	8.23E-07	5.18%
MIX	TARA_B100000686	1.16E-08	1.65E-06	0.70%
MIX	TARA_B100001123	7.38E-08	8.52E-06	0.87%
SRF	TARA_A100000164	6.21E-08	3.19E-07	19.47%
SRF	TARA_A100001015	1.47E-08	3.70E-08	39.77%
SRF	TARA_A100001234	1.04E-07	3.47E-07	29.94%
SRF	TARA_A200000113	2.66E-07	5.87E-06	4.53%
SRF	TARA_B000000475	7.17E-09	2.46E-08	29.17%
SRF	TARA_B000000532	2.58E-08	4.88E-08	52.82%
SRF	TARA_B000000565	7.64E-08	1.80E-07	42.51%
SRF	TARA_B100000003	1.47E-08	2.27E-08	64.82%
SRF	TARA_B100000066	5.90E-07	1.34E-06	44.18%
SRF	TARA_B100000123	7.75E-08	2.23E-07	34.78%
SRF	TARA_B100000161	5.30E-08	2.39E-07	22.16%
SRF	TARA_B100000212	2.71E-07	3.65E-07	74.21%
SRF	TARA_B100000242	2.12E-09	6.10E-08	3.48%
SRF	TARA_B100000282	1.69E-07	6.47E-07	26.11%
SRF	TARA_B100000401	4.80E-08	5.73E-08	83.79%
SRF	TARA_B100000424	1.15E-07	9.24E-07	12.45%
SRF	TARA_B100000459	2.82E-07	2.62E-06	10.76%
SRF	TARA_B100000475	1.18E-07	3.50E-06	3.37%
SRF	TARA_B100000497	2.88E-08	1.70E-07	16.91%
SRF	TARA_B100000513	3.14E-08	2.38E-06	1.32%
SRF	TARA_B100000578	1.17E-08	2.87E-07	4.08%
SRF	TARA_B100000925	1.93E-08	9.63E-08	20.04%
SRF	TARA_B100000963	2.29E-08	2.04E-06	1.12%
SRF	TARA_B100001057	1.00E-08	1.82E-06	0.55%
SRF	TARA_B100001063	2.92E-07	2.49E-06	11.73%
SRF	TARA_B100001093	7.83E-09	1.43E-07	5.48%
SRF	TARA_B100001109	1.11E-07	1.70E-06	6.53%
SRF	TARA_B100001115	7.10E-09	8.91E-07	0.80%
SRF	TARA_B100001121	1.14E-08	2.02E-07	5.64%
SRF	TARA_B100001540	6.13E-09	1.18E-07	5.19%
SRF	TARA_B100001564	2.60E-08	7.49E-08	34.71%
SRF	TARA_B100001758	4.07E-09	1.29E-08	31.65%
SRF	TARA_B100001769	5.99E-08	2.52E-07	23.81%
SRF	TARA_B100001989	1.30E-08	5.45E-07	2.39%
SRF	TARA_B100002019	2.96E-08	6.01E-07	4.93%
SRF	TARA_E500000075	1.36E-08	5.01E-07	2.71%
SRF	TARA_E500000178	5.01E-08	4.10E-07	12.23%
SRF	TARA_X000000263	9.10E-09	8.84E-07	1.03%
SRF	TARA_X000000950	2.61E-07	1.37E-06	19.02%
		Average		18.25%

Table S6 Motility-associated genes in FDHY-CJ and FDHY-03 annotated using KEGG pathway.

(a) FDHY-CJ

Gene ID	Gene name	Length	Description
ctg_00066	fliL	137	flagellar protein FliL
ctg_00138	mcp	439	methyl-accepting chemotaxis protein
ctg_00246	mcp	394	methyl-accepting chemotaxis protein
ctg_00247	cheY	121	two-component system, chemotaxis family, chemotaxis protein CheY
ctg_00248	cheA	697	two-component system, chemotaxis family, sensor kinase CheA
ctg_00249	cheW	167	purine-binding chemotaxis protein CheW
ctg_00250	aer	966	aerotaxis receptor
ctg_00251	cheR	276	chemotaxis protein methyltransferase CheR
ctg_00252	cheD	209	chemotaxis protein CheD
ctg_00253	cheB	348	two-component system, chemotaxis family, protein-glutamate methylesterase/glutaminase
ctg_00254	mcp	403	methyl-accepting chemotaxis protein
ctg_00255	cheY	121	two-component system, chemotaxis family, chemotaxis protein CheY
ctg_00256	cheA	653	two-component system, chemotaxis family, sensor kinase CheA
ctg_00257	cheW	167	purine-binding chemotaxis protein CheW
ctg_00258	tsr	768	methyl-accepting chemotaxis protein I, serine sensor receptor
ctg_00259	aer	969	aerotaxis receptor
ctg_00260	cheR	277	chemotaxis protein methyltransferase CheR
ctg_00261	cheD	205	chemotaxis protein CheD
ctg_00262	cheB	348	two-component system, chemotaxis family, protein-glutamate methylesterase/glutaminase
ctg_00312	mcp	636	methyl-accepting chemotaxis protein
ctg_00383	flgH	249	flagellar L-ring protein FlgH
ctg_00384	flgT	417	flagellar H-ring protein FlgT
ctg_00527	mcp	540	methyl-accepting chemotaxis protein
ctg_00543	cheY	356	two-component system, chemotaxis family, chemotaxis protein CheY
ctg_00597	aer	438	aerotaxis receptor
ctg_00622	mcp	539	methyl-accepting chemotaxis protein
ctg_00623	rpoD	610	RNA polymerase primary sigma factor
ctg_00670	mcp	674	methyl-accepting chemotaxis protein
ctg_00727	rpoN	493	RNA polymerase sigma-54 factor
ctg_00815	mcp	432	methyl-accepting chemotaxis protein
ctg_00883	cheY	331	two-component system, chemotaxis family, chemotaxis protein CheY
ctg_01034	mcp	635	methyl-accepting chemotaxis protein
ctg_01035	cheW	515	purine-binding chemotaxis protein CheW
ctg_01036	cheB	345	two-component system, chemotaxis family, protein-glutamate methylesterase/glutaminase
ctg_01038	cheR	284	chemotaxis protein methyltransferase CheR
ctg_01039	cheY	125	two-component system, chemotaxis family, chemotaxis protein CheY
ctg_01040	cheA	876	two-component system, chemotaxis family, sensor kinase CheA
ctg_01043	cheY	122	two-component system, chemotaxis family, chemotaxis protein CheY
ctg_01044	mcp	429	methyl-accepting chemotaxis protein
ctg_01249	cheV	319	two-component system, chemotaxis family, chemotaxis protein CheV
ctg_01301	mcp	540	methyl-accepting chemotaxis protein
ctg_01389	motA	253	chemotaxis protein MotA
ctg_01390	motB	309	chemotaxis protein MotB
ctg_01436	mcp	747	methyl-accepting chemotaxis protein
ctg_01518	flgO	221	flagellar H-ring protein FlgO
ctg_01519	flgO	254	flagellar H-ring protein FlgO
ctg_01520	flgO	241	flagellar H-ring protein FlgO
ctg_01584	mcp	639	methyl-accepting chemotaxis protein
ctg_01740	mcp	668	methyl-accepting chemotaxis protein
ctg_02717	cheY	266	two-component system, chemotaxis family, chemotaxis protein CheY
ctg_02856	motY	290	sodium-type flagellar protein MotY
ctg_02974	mcp	673	methyl-accepting chemotaxis protein
ctg_02981	cheW	163	purine-binding chemotaxis protein CheW
ctg_02982	cheW	268	purine-binding chemotaxis protein CheW
ctg_02984	cheB	376	two-component system, chemotaxis family, protein-glutamate methylesterase/glutaminase
ctg_02985	cheA	744	two-component system, chemotaxis family, sensor kinase CheA
ctg_02986	cheZ	251	chemotaxis protein CheZ

ctg_02987	cheY	123	two-component system, chemotaxis family, chemotaxis protein CheY
ctg_02988	fliA	243	RNA polymerase sigma factor FliA
ctg_02991	flhA	699	flagellar biosynthesis protein FlhA
ctg_02993	flhB	374	flagellar biosynthesis protein FlhB
ctg_02994	fliR	259	flagellar biosynthesis protein FliR
ctg_02995	fliQ	89	flagellar biosynthesis protein FliQ
ctg_02996	fliP	251	flagellar biosynthesis protein FliP
ctg_02997	fliO	150	flagellar protein FliO
ctg_02998	fliN	135	flagellar motor switch protein FliN
ctg_02999	fliM	352	flagellar motor switch protein FliM
ctg_03000	fliL	117	flagellar protein FliL
ctg_03001	fliK	771	flagellar hook-length control protein FliK
ctg_03002	fliJ	148	flagellar protein FliJ
ctg_03003	fliI	444	flagellum-specific ATP synthase [EC:7.4.2.8]
ctg_03004	fliH	260	flagellar assembly protein FliH
ctg_03005	fliG	346	flagellar motor switch protein FliG
ctg_03006	fliF	564	flagellar M-ring protein FliF
ctg_03007	fliE	111	flagellar hook-basal body complex protein FliE
ctg_03008	flrC	445	two-component system, response regulator FlrC
ctg_03010	flrA	487	sigma-54 dependent transcriptional regulator, flagellar regulatory protein
ctg_03011	fliC	264	hag`flagellin
ctg_03030	fliS	142	flagellar secretion chaperone FliS
ctg_03031	fliD	476	flagellar hook-associated protein 2
ctg_03033	fliC	279	hag`flagellin
ctg_03034	fliC	281	hag`flagellin
ctg_03035	fliC	281	hag`flagellin
ctg_03037	flgL	407	flagellar hook-associated protein 3 FlgL
ctg_03038	flgK	680	flagellar hook-associated protein 1
ctg_03039	flgJ	316	peptidoglycan hydrolase FlgJ
ctg_03040	flgI	370	flagellar P-ring protein FlgI
ctg_03041	flgH	226	flagellar L-ring protein FlgH
ctg_03042	flgG	262	flagellar basal-body rod protein FlgG
ctg_03043	flgF	247	flagellar basal-body rod protein FlgF
ctg_03044	flgE	461	flagellar hook protein FlgE
ctg_03045	flgD	228	flagellar basal-body rod modification protein FlgD
ctg_03046	flgC	140	flagellar basal-body rod protein FlgC
ctg_03047	flgB	144	flagellar basal-body rod protein FlgB
ctg_03048	cheR	276	chemotaxis protein methyltransferase CheR
ctg_03049	cheV	309	two-component system, chemotaxis family, chemotaxis protein CheV
ctg_03050	flgA	239	flagellar basal body P-ring formation protein FlgA
ctg_03051	flgM	109	negative regulator of flagellin synthesis FlgM
ctg_03052	flgN	143	flagellar biosynthesis protein FlgN
ctg_03053	flgP	192	outer membrane protein FlgP
ctg_03054	flgT	393	flagellar H-ring protein FlgT
ctg_03099	cheY	132	two-component system, chemotaxis family, chemotaxis protein CheY
ctg_03185	cheY	125	two-component system, chemotaxis family, chemotaxis protein CheY
ctg_03266	mcp	438	methyl-accepting chemotaxis protein
ctg_03301	cheY	121	two-component system, chemotaxis family, chemotaxis protein CheY
ctg_03302	cheC	194	chemotaxis protein CheC
ctg_03449	aer	521	aerotaxis receptor
ctg_03488	fliC	446	hag`flagellin
ctg_03518	motX	214	sodium-type polar flagellar protein MotX
ctg_03530	mcp	531	methyl-accepting chemotaxis protein
ctg_03707	cheX	154	chemotaxis protein CheX
ctg_03725	aer	527	aerotaxis receptor

(b) FDHY-03

Gene ID	Gene nam	Length	Description
gene0066	fliL	137	flagellar protein FliL
gene0161	mcp	493	methyl-accepting chemotaxis protein

gene0304	mcp	394	methyl-accepting chemotaxis protein
gene0305	cheY	121	two-component system, chemotaxis family, chemotaxis protein CheY
gene0306	cheA	697	two-component system, chemotaxis family, sensor kinase CheA
gene0307	cheW	167	purine-binding chemotaxis protein CheW
gene0308	mcp	966	methyl-accepting chemotaxis protein
gene0309	cheR	276	chemotaxis protein methyltransferase CheR
gene0310	cheD	209	chemotaxis protein CheD
gene0311	cheB	348	two-component system, chemotaxis family, protein-glutamate methylesterase/glutaminase
gene0312	mcp	403	methyl-accepting chemotaxis protein
gene0313	cheY	121	two-component system, chemotaxis family, chemotaxis protein CheY
gene0314	cheA	653	two-component system, chemotaxis family, sensor kinase CheA
gene0315	cheW	167	purine-binding chemotaxis protein CheW
gene0316	mcp	768	methyl-accepting chemotaxis protein
gene0317	mcp	969	methyl-accepting chemotaxis protein
gene0318	cheR	277	chemotaxis protein methyltransferase CheR
gene0319	cheD	205	chemotaxis protein CheD
gene0320	cheB	348	two-component system, chemotaxis family, protein-glutamate methylesterase/glutaminase
gene0370	mcp	636	methyl-accepting chemotaxis protein
gene0441	flgH	249	flagellar L-ring protein FlgH
gene0577	mcp	540	methyl-accepting chemotaxis protein
gene0646	mcp	438	methyl-accepting chemotaxis protein
gene0671	mcp	539	methyl-accepting chemotaxis protein
gene0672	rpoD	610	RNA polymerase primary sigma factor
gene0719	mcp	674	methyl-accepting chemotaxis protein
gene0776	rpoN	493	RNA polymerase sigma-54 factor
gene0864	mcp	432	methyl-accepting chemotaxis protein
gene1097	mcp	635	methyl-accepting chemotaxis protein
gene1098	cheW	515	purine-binding chemotaxis protein CheW
gene1099	cheB	345	two-component system, chemotaxis family, protein-glutamate methylesterase/glutaminase
gene1101	cheR	284	chemotaxis protein methyltransferase CheR
gene1102	cheY	125	two-component system, chemotaxis family, chemotaxis protein CheY
gene1103	cheA	876	two-component system, chemotaxis family, sensor kinase CheA
gene1106	cheY	122	two-component system, chemotaxis family, chemotaxis protein CheY
gene1107	mcp	429	methyl-accepting chemotaxis protein
gene1304	cheV	319	two-component system, chemotaxis family, chemotaxis protein CheV
gene1356	mcp	540	methyl-accepting chemotaxis protein
gene1443	motA	253	chemotaxis protein MotA
gene1444	motB	309	chemotaxis protein MotB
gene1505	mcp	747	methyl-accepting chemotaxis protein
gene1651	mcp	639	methyl-accepting chemotaxis protein
gene1820	mcp	668	methyl-accepting chemotaxis protein
gene2783	cheY	266	two-component system, chemotaxis family, chemotaxis protein CheY
gene2917	motY	290	sodium-type flagellar protein MotY
gene3118	cheW	163	purine-binding chemotaxis protein CheW
gene3119	cheW	268	purine-binding chemotaxis protein CheW
gene3121	cheB	376	two-component system, chemotaxis family, protein-glutamate methylesterase/glutaminase
gene3122	cheA	744	two-component system, chemotaxis family, sensor kinase CheA
gene3123	cheZ	251	chemotaxis protein CheZ
gene3124	cheY	123	two-component system, chemotaxis family, chemotaxis protein CheY
gene3125	fliA	243	RNA polymerase sigma factor FliA
gene3128	flhA	699	flagellar biosynthesis protein FlhA
gene3130	flhB	374	flagellar biosynthesis protein FlhB
gene3131	fliR	259	flagellar biosynthesis protein FliR
gene3132	fliQ	89	flagellar biosynthesis protein FliQ
gene3133	fliP	251	flagellar biosynthesis protein FliP
gene3134	fliO	150	flagellar protein FliO/FliZ
gene3135	fliN	135	flagellar motor switch protein FliN
gene3136	fliM	352	flagellar motor switch protein FliM
gene3137	fliL	179	flagellar protein FliL
gene3138	fliK	771	flagellar hook-length control protein FliK

Table S7 Comparison of the number of motility-associated genes between FDHY-CJ and FDHY-03 annotated using KEGG pathway.

Gene name	FDHY-CJ	FDHY-03
aer	5	1
cheA	4	4
cheB	4	4
cheC	1	1
cheD	2	2
cheR	4	4
cheV	2	2
cheW	5	5
cheX	1	1
cheY	11	8
cheZ	1	1
flgA	1	1
flgB	1	1
flgC	1	1
flgD	1	1
flgE	1	1
flgF	1	1
flgG	1	1
flgH	2	2
flgI	1	1
flgJ	1	1
flgK	1	1
flgL	1	1
flgM	1	1
flgN	1	1
flgO	3	0
flgP	1	1
flgT	2	0
flhA	1	1
flhB	1	1
fliA	1	1
fliC	5	5
fliD	1	1
fliE	1	1
fliF	1	1
fliG	1	1
fliH	1	1
fliI	1	1
fliJ	1	1
fliK	1	1
fliL	2	2
fliM	1	1
fliN	1	1
fliO	1	1
fliP	1	1
fliQ	1	1
fliR	1	1
fliS	1	1
flrA	1	1
flrC	1	1
mcp	17	24
motA	1	1
motB	1	1
motX	1	1
motY	1	1
rpoD	1	1
rpoN	1	1
tsr	1	0
